# Evaluation of the role of repeated inferior vena cava sonography in estimating first 24 h fluid requirement in resuscitation of major blunt trauma patients in emergency department Suez Canal University Hospital

**DOI:** 10.1186/s12873-024-01033-7

**Published:** 2024-07-16

**Authors:** Rasha Mahmoud Ahmed, Bassant Sayed Moussa, Mohamed Amin Ali, Aml Ibrahiem Sayed Ahmed Abo El Sood, Gouda Mohamed El Labban

**Affiliations:** 1 Faculty of medicine, Suez Canal University, Ismailia, Egypt; 2 General SurgeryFaculty of medicine, Suez Canal University, Ismailia, Egypt

**Keywords:** IVC, Repleted, Hypovolemic shock

## Abstract

**Introduction:**

The assessment of hemodynamic status in polytrauma patients is an important principle of the primary survey of trauma patients, and screening for ongoing hemorrhage and assessing the efficacy of resuscitation is vital in avoiding preventable death and significant morbidity in these patients. Invasive procedures may lead to various complications and the IVC ultrasound measurements are increasingly recognized as a potential noninvasive replacement or a source of adjunct information.

**Aimof this study:**

The study aimed to determine if repeated ultrasound assessment of the inferior vena cava (diameter, collapsibility (IVC- CI) in major trauma patients presenting with collapsible IVC before resuscitation and after the first hour of resuscitation will predict total intravenous fluid requirements at first 24 h.

**Patients & methods:**

The current study was conducted on 120 patients presented to the emergency department with Major blunt trauma (having significant injury to two or more ISS body regions or an ISS greater than 15). The patients(cases) group (shocked group) (60) patients with signs of shock such as decreased blood pressure < 90/60 mmHg or a more than 30% decrease from the baseline systolic pressure, heart rate > 100 b/m, cold, clammy skin, capillary refill > 2 s and their shock index above0.9. The control group (non-shocked group) (60) patients with normal blood pressure and heart rate, no other signs of shock (normal capillary refill, warm skin), and (shock index ≤ 0.9). Patients were evaluated at time 0 (baseline), 1 h after resucitation, and 24 h after 1st hour for:(blood pressure, pulse, RR, SO2, capillary refill time, MABP, IVCci, IVCmax, IVCmin).

**Results:**

Among 120 Major blunt trauma patients, 98 males (81.7%) and 22 females (18.3%) were included in this analysis; hypovolemic shocked patients (60 patients) were divided into two main groups according to IVC diameter after the first hour of resuscitation; IVC repleted were 32 patients (53.3%) while 28 patients (46.7%) were IVC non-repleted. In our study population, there were statistically significant differences between repleted and non-repleted IVC cases regarding IVCD, DIVC min, IVCCI (on arrival) (after 1 h) (after 24 h of 1st hour of resuscitation) ( *p-*value < 0.05) and DIVC Max (on arrival) (after 1 h) (*p-*value < 0.001). There is no statistically significant difference (*p-*value = 0.075) between repleted and non-repleted cases regarding DIVC Max (after 24 h).In our study, we found that IVCci0 at a cut-off point > 38.5 has a sensitivity of 80.0% and Specificity of 85.71% with AUC 0.971 and a good 95% CI (0.938 – 1.0), which means that IVCci of 38.6% or more can indicate fluid responsiveness. We also found that IVCci 1 h (after fluid resuscitation) at cut-off point > 28.6 has a sensitivity of 80.0% and Specificity of 75% with AUC 0.886 and good 95% CI (0.803 – 0.968), which means that IVCci of 28.5% or less can indicate fluid unresponsiveness after 1st hour of resuscitation. We found no statistically significant difference between repleted and non-repleted cases regarding fluid requirement and amount of blood transfusion at 1st hour of resuscitation (*p-*value = 0.104).

**Conclusion:**

Repeated bedside ultrasonography of IVCD, and IVCci before and after the first hour of resuscitation could be an excellent reliable invasive tool that can be used in estimating the First 24 h of fluid requirement in Major blunt trauma patients and assessment of fluid status.

## Introduction

Hypovolemic shock is a circulatory malfunction associated with inadequate tissue perfusion that leads to multiorgan failure. Since it results from either blood loss or extracellular fluid loss, quick onset hypovolemia [1] is usually the cause. Blood loss-related hypovolemic shock is known as hemorrhagic shock [2].

Hemorrhagic shock is typically the primary cause of shock in trauma victims, while numerous other conditions can also contribute to shock. Hemorrhage accounts for 30 to 40% of trauma-related deaths, of which 33 to 56% happen in the prehospital phase and over 40% happen in the first 24 h [3]. This means that it can be lethal very quickly. Hematocrit levels, biochemical markers, physical examination results, and other conventional measures are used to identify hypovolemia, but are not precise markers or trustworthy since they can be deemed normal when the body's compensatory mechanisms kick in, which could cause delays in the identification of volume loss [4].

While tachycardia is a symptom of acute fluid loss, its sensitivity and specificity are insufficient for diagnosis or monitoring because it can be altered by a variety of internal and external signals [5].

As A central line is an invasive procedure with potential complications (venous thrombosis, infection, pneumothorax, arterial puncture) during or after the procedure [6] and poor predictive value [4], measurement of CVP is not practically used in hypovolemic patients admitted to the emergency department (ED).

Focused assessment with sonography for trauma (FAST) has shown to be an effective way to identify bleeding sites in trauma patients using sonography. FAST took an average of 4 min per patient and was used successfully as a primary screening procedure at the hospital's entrance for traumatized mass casualty patients [7]. However, it provides no information regarding the patient's hemodynamic status, blood loss, amount lost, or response to resuscitation [8].

As such, we require techniques for early detection of hypovolemia and accurate follow-up. The use of ultrasonography in critical care has grown in recent years due to technological advancements. The IVC is widely acknowledged as a trustworthy metric for assessing hemodynamic conditions [7]. Approximately 80% of the venous return to the right atrium is carried by the high-capacitance Inferior Vena Cava vein (IVC). The IVC pathway is exclusively abdominal and is influenced by the right atrial pressure [6], intravascular volume [9], and intraabdominal pressure. When the body experiences volume loss, the compensatory vasoconstrictor reaction has little effect on the IVC diameter [10]. The noninvasive, quick diagnosis, and low cost of sonographic measurement of IVC diameter make this method a valuable tool for determining fluid requirements [12] and evaluating volume status in critically ill patients [11].

## Aim of the study

To evaluate the effectiveness of Repeated IVC Sonography before and after the first hour of resuscitation in estimating the First 24 h of fluid requirement in Major blunt trauma patients.

### Study objectives

#### Primary objective

—To estimate and predict the First 24-h fluid requirement using repeated IVC Sonography before and after the first hour of resuscitation. 

Secondary objectives

#### Secondary objectives

—To assess the role of IVC in rapidly detecting early shock stages.

### Patient and methods

#### Study type

This study was conducted as a prospective cohort study.

#### Study sites

i) Patients’ recruitment: The major blunt trauma patients (having a significant injury to two or more ISS (Injury Severity Score) body regions or an ISS greater than 15) [13] were recruited from the emergency department, Suez Canal University Hospital, Ismailia and this study was carried out for one year (from February 2022 to February 2023),shocked patients were managed in our ED Resucitation Room and the length of stay accordindg to patient condition and resucitation efforts.

#### Study population

A Patients group: Sixty of both genders aged 18–60 years old. Major Blunt trauma patients with signs of shock such as decreased blood pressure < 90/60 mmHg or a more than 30% decrease from the baseline systolic pressure, heart rate > 100 b/m, cold, clammy skin, capillary refill > 2 s [2] and (shock index above 0.9) attended Suez Canal University Hospital.
B Control group: Sixty of both genders aged 18–60 years old Major Blunt trauma patients with normal blood pressure (≥ 90/60 mmHg) and average heart rate, no other signs of shock (normal capillary refill, warm skin) and (shock index ≤ 0.9) attended Suez Canal University Hospital.

#### Inclusion criteria

Major blunt traumatic patients who came to ER Suez Canal Hospital were admitted after stabilization to the Inpatient ward or ICU.

#### Exclusion criteria

## Subjects were excluded from the study if they had any of the following:

- Age below 18 and more than 60.

- Cardiopulmonary resuscitation on arrival.

- Transferred from another hospital (as fluid will be given before IVC sonography is done).

- Traumatic brain injury or cervical spine injury alone (as IVC will not collapse and no hypovolemia).

- Severe injury to the lower chest and upper abdominal wall, subcutaneous emphysema (they will hinder the IVC visualization, and pain in this area will hinder repeated clear sonography).

- Morbid obesity. (as no clear images).

- Contraindicating fluid challenges, such as cardiac insufficiency and Renal failure.

- Tricuspid regurge, right-sided heart disease, obstructive lung disease, and portal hypertension, affect IVC diameter as an increase in the Right atrial pressure.

- Mechanically ventilated patients as in positive pressure ventilation, respiratory changes in IVCD become reversed.

- Pregnant women. (high intra-abdominal pressure).

## Statistical plan

According to the following equation, the estimated sample size will be 60 subjects in each group.

$$n= {\left[\frac{{\text{Z}}_{\propto /2}+{\text{Z}}_{\upbeta }}{\frac{1}{2}\text{log}\frac{1+r}{1-r}}\right]}^{2}+3$$

***n*** = required sample size.

**Zα/2** = 1.96 (The critical value that divides the central 95% of the Z distribution from the 5% in the tail).

**Zβ** = 0.84 (The critical value that separates the lower 20% of the Z distribution from the upper 80%) **r** = correlation [15].

So, *n* = 54 subjects per group, we will add 10% (≃ 6) to compensate for non-responders, and the sample size will be 60 subjects in each group.

### Data management

- Data was collected and coded, then entered as a spreadsheet using Microsoft Excel for Windows Office 2010.

- Gathered data was processed using SPSS version 22.0.0.0.

- Quantitative data was expressed as means ± SD, while qualitative data was expressed as numbers and percentages (%).

- Sensitivity, specificity, accuracy, positive and negative predictive values were calculated.

- Student t-test was used to test the significance of the difference for quantitative variables, and Chi-Square was used to test the significance of the difference for qualitative variables.

- A probability value (*p-*value) < 0.05 was considered statistically Significant.

### Ethics consideration

- The study protocol was approved by the Research Ethics Committee of the Faculty of Medicine Suez Canal University before starting the fieldwork.

- Written informed consent was obtained from all the participants before taking any data or doing any investigations.

- The consent contained:

- Explanation of the study aimed to be understood by the common people.

- No harmful maneuvers was performed or used.

- All data were confidential and weren’t used outside this study frame without the patient's approval.

The researcher's phone number and all possible communication methods were given to the participants to return at any time for any explanation.

- Patients and volunteers had the right to refuse to participate or withdraw from the research at any time with no consequent resentment.

- All participants were be informed of the result of the study.

- Permission and signature of the participants were taken.

All participants' samples weren’t shipped outside the country, were used in this research only, and eliminated after the research is finished.

Data was collected in a pre-organized data sheet ( MDH Statewide Trauma System, 2015) by the researcher from patients fulfilling inclusion and exclusion criteria.

### Socio-demographic data

-  Patient's file number.
- Images were stored through at least two respiratory cycles. The (Diameter Inferior Vena Cava at Maximum of expiration) (DIVC max) and Diameter Inferior Vena Cava at Minimum of inspiration (DIVC min) IVC dimensions were obtained by measuring the vein lumen during a regular breathing cycle from one interior wall to the opposite interior wall. Inferior Vena Cava Collapsability (IVCCI) was calculated using the following formula
-  Patient personal data: Age and Sex.
-  Trauma data Time, mechanism of injury, anatomical site, associated injuries, clinical presentation, and event.
-  Present illness history: Onset, course, duration of the symptoms, and history of previous investigations.
-  Allergy, Drug history, Last meal, Past history.

### Clinical examination

1. Vital signs: pulse, blood pressure, respiratory rate, body temperature, and oxygen saturation, pain severity on visual analog scale if conscious patient.
2. Initial assessment of ABCDE (airway and cervical spine control, breathing, circulation, dysfunction of central nervous system, and exposure) and Glasgow coma scale.
3. Trauma sheet data., ISS: site of injury, such as the head, Neck, chest, abdominal, Back, pelvic, and extremities.
4. Assessment of the condition of the patients either stable or unstable which will determine the needed investigations and plane of management.

### Investigations

Included.

1. Laboratory investigations, as complete blood count (normal values of blood typing and cross match and coagulation profile (platelet count, prothrombin time *(PT*), *partial* thromboplastin time *(*PTT*)*, International normalized ratio of prothrombin time (INR), base excess(BE): normal range (-2 to + 2 mEq/L) [16].

s. Lactate (Normal lactate levels are less than two mmol/L), Arterial Blood Gases.

Total amount of red blood cells(RBCs), fresh frozen plasma (FFP), and platelet transfusion within the first 24 h from injury were recorded.

2. Radiographic investigations**,** as Plain chest x-ray, Upper extremity (hand- wrist – forearm (ulna, radius)-arm –shoulder), lower extremity x-ray [pelvis, both hips, femur, leg ( tibia, fibula), ankle and foot] and FAST and IVC Sonography. Brain Computed Tomography may be needed in some stable cases (who were unconscious or neurologically not assessable for other reasons). CT scans of other body regions (e.g. chest, abdomen) were done if lesions were suspected after clinical examination or FAST.

The major blunt trauma patients (having a significant injury to two or more ISS body regions or an ISS greater than 15) [13] were divided into two groups: The patient’s group (shocked group) (60) patients with signs of shock such as decreased blood pressure < 90/60 mmHg or a more than 30% decrease from the baseline systolic pressure, heart rate > 100 b/m, cold clammy skin, capillary refill > 2 s [2] and their shock index above0.9.

The control group (the non-shocked group) (60) patients with normal blood pressure and heart rate, no other signs of shock (normal capillary refill, warm skin), and (a shock index ≤ 0.9).

In the first hour of resuscitation, the patients (unstable shocked) were given 1 L of crystalloid solution for fluid resuscitation ( according to recent ATLS guidelines). If the patient was still unstable after 1 Liter of fluid or had ongoing blood loss, O-negative blood transfusion was administered and type-specific blood was ordered and the trauma team activated massive transfusion protocol according to recent ATLS Guidelines even if normal hemoglobin [17].

The evaluations were performed by an emergency and radiologist specialists on arrival ( the emergency specialist took ER ultrasound training courses, application 3 years in ER) All examinations were performed in the supine position with the ultrasound transducer placed in a subxiphoid location during quiet passive respiration, using either a portable US machine (Butterfly iQ/iQ + ™) or Phillips HD11EXm.

A linear probe with a frequency ranging from 3.5–7.5 MHZ and Sagittal sections of the IVC behind the liver were imaged. The IVC diameter was measured approximately 2 cm distal to the IVC-hepatic vein junction and 2 cm away from the Right Atrium (RA inlet), where the anterior and posterior walls of the IVC are visible and parallel to each other.

- Images were stored through at least two respiratory cycles. The (Diameter Inferior Vena Cava at Maximum of expiration) (DIVC max) and Diameter Inferior Vena Cava at Minimum of inspiration (DIVC min) IVC dimensions were obtained by measuring the vein lumen during a regular breathing cycle from one interior wall to the opposite interior wall. Inferior Vena Cava Collapsability (IVCCI) was calculated using the following formula

$$\mathrm{IVCCI}=\left(\mathrm{dIVC}\;\mathrm{expiration}\;-\;\mathrm{dIVC}\right)\;/\;\mathrm{dIVC}\;\mathrm{expiration}\;\times\;100$$

IVC sonography was assessed in both groups:

- On arrival (before resuscitation).
- After 60 min of initial resuscitation.
- and after 24 h of admission.

The shocked group after 1 h of resuscitation were divided into two groups (IVC non -Repleted) and (IVC Repleted) group as (if there is still an IVC diameter of < 10 mm after the first hour of resuscitation they are called (the IVC non -Repleted) group.

And if the IVC diameter of ≥ 10 mm after the first hour of resuscitation) they are called the (IVC Repleted) group [18].

In this study, we predicted that after the first hour of resuscitation (the IVC non -Repleted) group needed more than 1–2 ml/kg/hour in the first24 hours (more than 2400 ml fluid), and the (IVC Repleted) group we predicted that after the first hour of resuscitation, they will need the average 1-2 ml/kg/hr. (average 2400 ml fluid) or less in the first 24 h. Patients were clinically assessed and managed as per the ABC protocol.

### Fate at Emergency Room

Fate of the patient was recorded whether:

- had surgical intervention.
- Admitted to inpatient under observation.
- Admitted to the intensive care unit.
- Died.

## Results

The datasets generated and/or analysed during the current study are available in the [data sheet] repository and uploaded as separate files.

The total number of patients with different presentations who visited the ED from August 10, 2019, to March 09, 2020, was 28,301. Among them, 1,492 (5.3%) were patients with trauma. Of the 1,492 patients with trauma, 1,034 (69.3%) had minor injuries and did not meet the polytrauma criterion, and only 458 (30.7%) had poly-trauma. Therefore, the total incidence of trauma among all cases in the ED was 5.3%; however, this rate increased to 9.3%, especially in the weekends. Furthermore, the total number of cases admitted to SCUH during the study period was 7,509 (26.5%) of the 28,301 patients who visited the ED, among whom 762 (50.1%) were trauma cases; therefore, the total incidence of trauma admissions among the total admission cases that visited the ED was 10.2%. The total number of patients with polytrauma without life-threatening injuries was 333, and the total number of patients of poly-trauma with life-threatening injuries was 125.

The mean age in our study showed a Statistically significant difference between the two groups (case and control) as mean age ± SD was 32.13 ± 12.75 in unstable hypovolemic patients while in the control mean age ± SD 39.02 ± 13.52 (*p-*value = 0.008) as mentioned in Table 1.

**Table 1:** Comparisons of demographic data and ISS as regard IVC status

		**IVC status**				**Stat. test**	***P***-**value**
		**Non-repleted (N = 28)**	**Repleted (n = 32)**				
**Age (years)**	**Mean ± SD**	32.3 ± 13.3		32.4 ± 11.7		T = 0.004	0.997 NS
	**Min – Max**	18—55		18—47			
	**Median (IQR)**	27 (21—44)		32 (21—43)			
**Age groups**	**18 – 29 ys**	15	53.6%	12	37.5%	X^2^ = 4.01	0.260 NS
	**30 – 39 ys**	4	14.3%	10	31.3%		
	**40 – 49 ys**	5	17.9%	8	25%		
	**50 – 60 ys**	4	14.3%	2	6.3%		
**Sex**	**Male**	23	82.1%	24	75%	X^2^ = 0.44	0.503 NS
	**Female**	5	17.9%	8	25%		
**ISS**	**Mean ± SD**	35 ± 8.5		32.8 ± 8.2		T = 0.97	0.333 NS
	**Min – Max**	25—45		25—57			
	**Median (IQR)**	33 (29—42.75)		29 (27—35.5)			
**Anatomical site**	**Head and neck**	2	7.1%	4	12.5 %	0.47	0.490 NS
	**Face**	2	7.1%	4	12.5 %	0.47	0.490 NS
	**Chest**	20	71.4%	24	75 %	0.097	0.755 NS
	**Abdomen**	26	92.8%	29	90.6 %	0.4	0.997 NS
	**Extremity**	27	96.4%	30	93.8 %	0.22	0.635 NS

T: Independent sample T test. S: *p-*value < 0.05 is considered significant. X^2^: Chi-square test. NS: *p-*value > 0.05 is considered non-significant

This Table (1) shows:

• No statistical significant difference (***p***-value > 0.05) between repleted and non-repleted cases regarding age, age groups, sex,ISS and anatomical site of injury

Our study showed no statistically significant difference (*p-*value = 0.233) between studied groups (Cases and control) as regards GCS. A statistically significant difference (*p-*value < 0.001) in the Cases group when compared with the control group regarding to (Respiratory rate(R.R), Heart Rate (H.R) and shock index, systolic Blood Pressure (SBP), Diastolic Blood Pressure (DBP), and Mean Arterial Blood Pressure (MAP)) (on arrival, after 1 h, and after 24 h of resuscitation).

**Fig. 1:**
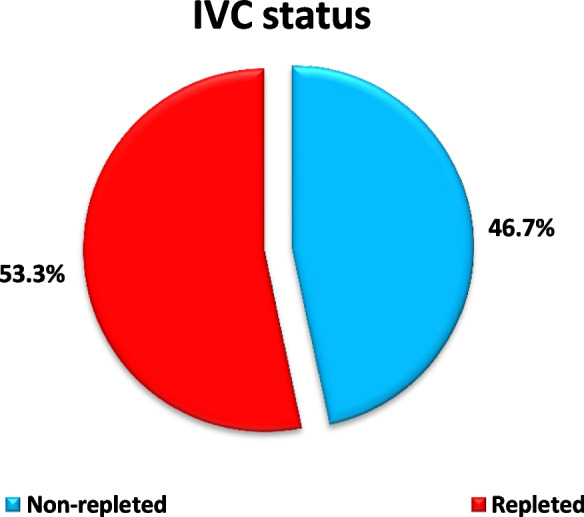
Shows that There were 28 patients (46.7%) non-repleted IVC and 32 patients (53.3%) repleted in cases group

Among 120 Major blunt trauma patients 98 males (81.7%) and 22 females (18.3%) included in this analysis, hypovolemic shocked patients (60 patients) were divided into two main groups according to IVC diameter after the first hour of resuscitation; repleted32 patients (53.3%) while 28 patients (46.7%) were IVC non-repleted as shown in Fig. 1.

In our study population, there was a statistically significant difference between repleted and non-repleted cases regarding IVCD, DIVC min, (after 1 h) (after 24 h of 1st hour of resuscitation) ( *p-*value < 0.05) and DIVC Max (on arrival) (after 1 h) (*p-*value < 0.001).

No statistically significant difference (*p-*value = 0.075) between repleted and non-repleted cases as regards DIVC Max (after 24 h).

We found no statistically significant difference between repleted and non-repleted cases as regards fluid requirement and amount of blood transfusion at 1st hour of resuscitation (*p-*value = 0.104). There is a statistically significant difference between repleted 5( 15.6%) and non-repleted 24 patients ( 85.7%) cases regarding blood transfusion (24 h after 1st hour of resuscitation) (*p-*value < 0.001), amount of blood transfusion (24 h after 1st hour of resuscitation) as in non-repleted patients Mean ± SD 1700 ± 1082)ml while Mean ± SD in repleted patients was(550 – 750) (*p-*value = 0.045), percentage of fluid requirement > 2400 ml (24 h) as 24 (86%) of IVC non repleted were FR + ve patients while in repleted patients only 4 (12.5%) FR + ve patients (*p-*value < 0.001) and total fluid requirement (24 h after 1st hour of resuscitation) as in non-repleted IVC Mean ± SD 3571.4 ± 705.4 ml while in repleted 2143.7 ± 397.9 ml (*p-*value < 0.001) as mentioned in Table 2.

**Table 2:** Description of fluid requirement in studied group

				**Cases group (N = 60)**	
**Fluid requirement (1st hour)**		**Mean ± SD**		1000 ± 0.0	
		**Min – Max**		1000 – 1000	
**Blood transfusion (1st hour)**		**No**		16	26.7%
		**Yes**		44	73.3%
**Blood transfusion (1st hour)**		**Mean ± SD**		668.2 ± 253.5	
		**Min – Max**		400 – 1150	
		**Median (IQR)**		550 (550 – 1150)	
**Blood transfusion (24 h)**		**No**		31	51.7%
		**Yes**		29	48.3%
**Blood transfusion (24 h)**		**Mean ± SD**		1556.3 ± 1081.9	
		**Min – Max**		550 – 3650	
		**Median (IQR)**		850 (550 – 2750)	
**Fluid requirement (FR)(24 h) > 2400 ml**		**FR –ve**		32	53.3%
		**FR + ve**		28	46.7%
		**Cases (N = 60)**	**Control (N = 60)**	**T**	***P*** **-value**
**Total fluid requirement (after 24 h)**	**Mean ± SD**	2810 ± 909.3	1595 ± 494.8	**9.09**	** < 0.001 S**
	**Min – Max**	1500—4350	1000 – 2200		
	**Median (IQR)**	2250 (2000—4000)	2000 (1000—2000)		

All studied patients of cases (shocked) group required fluid at the 1st hour of resucitation (1000) ml. There were 44 patients (73.3%) required blood transfusion at the 1st hour of resucitation (still hypotensive not responding to initial crystalloid given according to recent ATLS guidlines). There were 31 patients (51.7%) required blood transfusion after 24 h. The mean 24-h blood transfusion was 1556.3 ± 1081.9. There were 28 patients (46.7%) who required fluid > 2400 ml after 24 h. Also, this table shows the significant statistical difference between studied groups (shocked &non-shocked group) (*p-*value < 0.001) as regards to total fluid requirement after 24 h of the first hour of resuscitation)

**Fig. 2:**
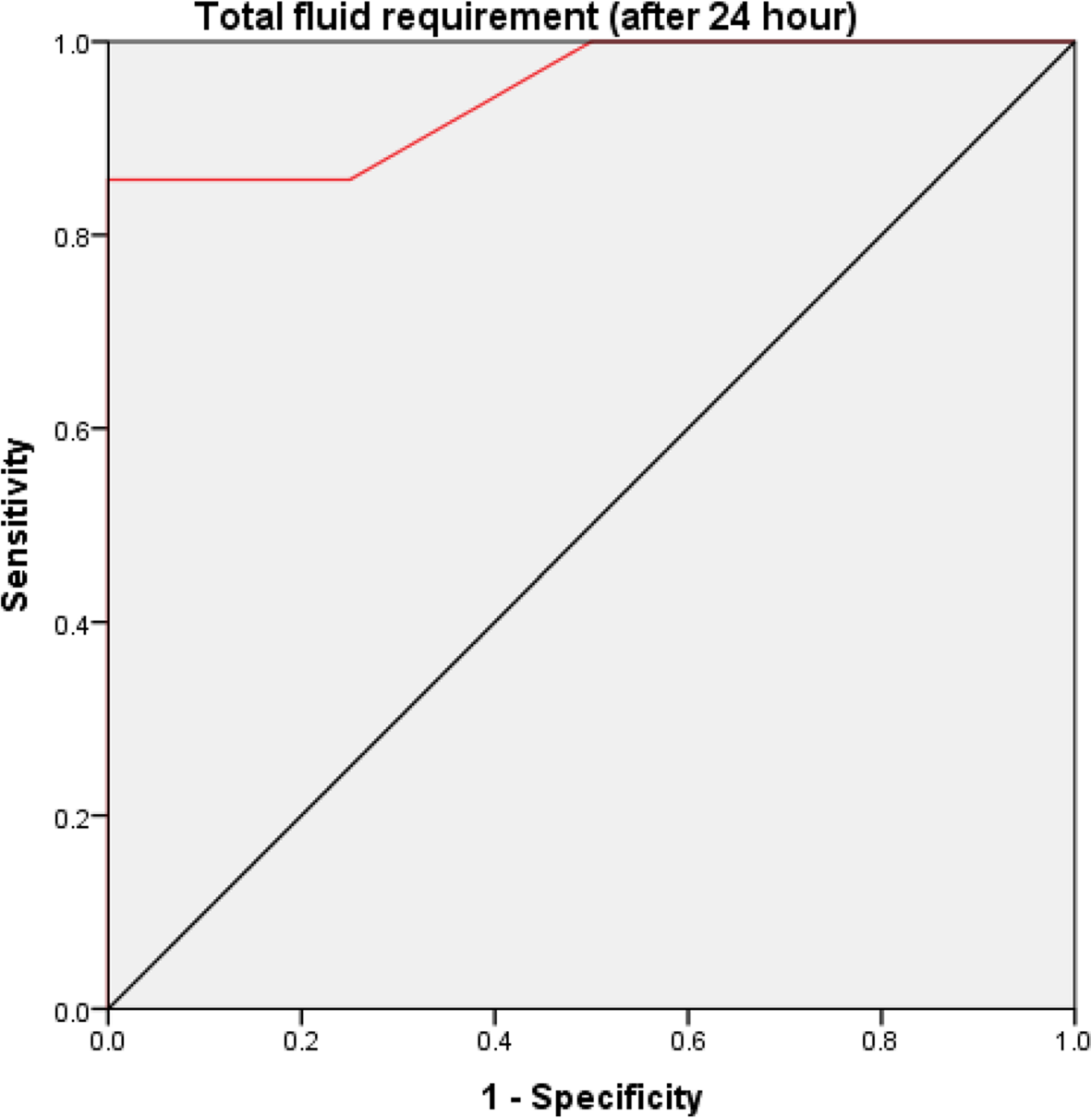
ROC curve between repleted and non-repleted patients as regard total fluid requirement after 24 h

Total fluid requirement (after 24 h) can be used to discriminate between depleted and non-depleted patients at a cutoff level of 2200, with 100% sensitivity, 87.5% specificity, 88.9% positive predictive value (PPV) and 100% negative predictive value (NPV) (AUC = 0.94 & *p-*value < 0.001) as shown in Fig. 2.

In our study, we found that IVCci0 at a cut-off point > 38.5 has a sensitivity of 80.0% and Specificity of 85.71% with Area under curve (AUC) 0.971 and a good 95% CI (0.938 – 1.0) which means that IVCci of 38.6% or more can indicate fluid responsiveness. We also found that IVCci 1 h (after fluid resuscitation) at cut-off point > 28.6 has a sensitivity of 80.0% and Specificity of 75% with AUC 0.886 and good 95% CI (0.803 – 0.968) which means that IVCci of 28.5% or less can indicate fluid unresponsiveness after 1st hour of resuscitation as mentioned in Table 3.

**Table 3:** Diagnostic performance of IVCCI in discrimination of IVC status (repleted vs non-repleted)

**IVCCI (%)**	**Cut off**	**AUC**	**Sensitivity**	**Specificity**	**PPV**	**NPV**	**p-value**
**On arrival**	>38.5	0.971	80.0	85.71	80.0	85.7	**< 0.001**
**1 hour**	>28.6	0.886	80.0	75	70.7	83.3	**< 0.001**
**24 hour**	**12.9**	**0.85**	**87.5%**	**88.9%**	**100**	**100**	**< 0.001**

*PPV* positive predictive value, *NPV* negative predictive value, *AUC* Area under curve

Using roc curve, it was shown that:

**• IVCCI (on arrival)** can be used to discriminate between repleted and non-repleted patients at a cutoff level of 38.5, with 80% sensitivity, 85.71% specificity, 80.0% PPV and 85.7% NPV **(AUC = 0.96 &** ***p*-value < 0.001)**

**• IVCCI (1 hour)** can be used to discriminate between repleted and non-repleted patients at a cutoff level of 28.6, with 0% sensitivity, 75% specificity, 70.7% PPV and 83.3% NPV **(AUC = 0.98 &** ***p*-value < 0.001)**

**• IVCCI (24 hour)** can be used to discriminate between repleted and non-repleted patients at a cutoff level of 12.9, with **87.5%** sensitivity, **88.9%** specificity, 100% PPV and 100 % NPV **(AUC = 0.85 &** ***p*-value < 0.001)**

## Discussion

Our goal was to ascertain whether repeat IVC sonography was useful in calculating the initial 24-h fluid demand in major blunt trauma patients, both before to and following the first hour of resuscitation.

The current study involved 120 patients with major blunt trauma and were brought to the emergency room (meaning they had sustained substantial injuries to two or more ISS body regions, or an ISS more than 15).

There was a statistically significant decrease in age in the Cases (shocked) group with mean ± SD( 32.4 ± 12.4) when compared with control, indicating that gender was male-dominated in our study with 98 males (81.7%) and 22 females (18.3%) with mean ± SD (39.01 ± 13.5) (*p-*value = 0.008).

Moreover, there were an age-related statistically significant difference (*p-*value = 0.003) between the two study groups (Cases and Control). Ages between 18 and 39 were the most common. There were 27 patients (45%) aged 18 to 29 years, 14 patients (23.3%) aged 30 to 39 years, and 15 patients (25%), aged 18 to 29 years, (33.3%) in our control group.

Elbaih and Housseini (2018) used the RUSH rapid ultrasonography in shock and hypotension protocol for early diagnosis of various kinds of shock to examine the reason of unstability in polytrauma patients. A total of 100 polytrauma patients were included in this study. There were 25 females (25%) and 75 males (75%) in all. The mean age of patients with polytrauma (19) was 27.5 ± 17.8 years, which is consistent with our research.

In 2023, Cholo et al. conducted an investigation into 1,073 motorcycle crash injury cases, all of which involved males in all categories of hospital attendance across all age groups (P < 0.001), with a mean age ± SD of 29.6 ± 12.19 years (20), which is consistent with our findings. Their goal was to examine the burden of trauma to society.

In 2018, de Vries and colleagues conducted an analysis of the 6-year Dutish trauma registry database. Of the 13,207 patients (52.2%) who were classified as polytrauma patients (group ages 18–59), their mean age ± SD was (40.1 ± 12.8). In this category, there were 25.2% more women than men.

Aism et al. in 2023 investigated a total of 1645 trauma patients who were admitted to the hospital they found that 24.5% had high Shock index (SI). The mean age was 39.2 ± 15.2 y, and most were males (91%). Patients with high SI were younger, and had sustained severer injuries [22] which agrees with our study.

In 2020, Hatton and colleagues conducted a study to examine the correlation between occult hypoperfusion and trauma outcomes. Among the 3,126 trauma patients, 808 were elderly patients. Contrary to our investigation, shock rates (33% and 31%) were comparable in younger and older individuals [23].

A study by Mehmood and Shafiq from 2019 involved 124 individuals who had been hospitalized for longer than 24 h after being involved in a traffic accident. Males made up the bulk of the injured patients (88%) and those between the ages of 20 and 40 (53.2%). Our study is further supported by the statistically significant (*p* = 0.016) difference observed between the various age groups [24].

Driving while intoxicated, abusing drugs or alcohol, or engaging in other psychoactive substances could all be contributing factors to the high rate of rear-end collisions (RTAs) among adult youth (ages 20 to 40). Young adult polytrauma causes significant harm and financial strain on the nation and family.

The study found a highly statistically significant difference (*p-*value < 0.001) in the Mean Injury Severity Score (ISS) between the unstable hypovolemic patients' 33.8 ± 8.4 and the control group's 22.8 ± 5.4.

Our research indicated that there was no statistically significant difference between the study groups regarding head and neck injuries (*p-*value = 0.191) based on the anatomical site of injury, but there was a statistically significant difference between the groups regarding chest affection, extremity, and abdomen (*p-*values 0.003, 0.004, < 0.001).

The findings of Jávor et al.'s [17] investigation of 156 hypovolemic shocked individuals are consistent with our findings. Of these patients, 84 (53.9%) had affection in the thorax, and 75 (48.1%) had affection in the extremities. Their ISS had a median of 29 with an IQR (20–34) [17].

In line with our findings, Abd EL-Atiff et al. [25] discovered that blunt trauma most frequently affected the extremities and pelvis (72%–25). In 2019, Kim et al. looked at 628 trauma patients in total, separating them into groups: survivors and non-survivors. The survival group's mean ± SD was 12.44 ± 11.20, whereas the non-survival group's mean ± SD was 28.15 ± 14.01, indicating a substantial statistical difference between the two groups (p < 0.001). Mortality in the chest (p < 0.001) and extremities (*p* = 0.021) was substantially correlated with the site of injury [26] which is consistent with our research.

The results of our study demonstrated a statistically significant difference (*p-*value < 0.001) between the studied groups (Cases and Control) with respect to RR, pulse, shock index (on arrival), (after 1 h), (after 24 h), as well as SBP, DBP, and MAP (on arrival, after 1 h, and after 24 h). also significant difference between studied groups (Cases and control) regarding Hemoglobin, Hematocrit, base deficit ( on arrival and after 1st hour of resuscitation) and PH (on arrival) (*p-*value < 0.001).

There was no statistically significant difference between the studied groups in our study (Cases and control) regarding GCS on arrival and PH after 1 h of resuscitation ( (*p-*value 0.164, 0.161).

In terms of RR, pulse, shock index (on arrival), (after 1 h), (after 24 h), as well as SBP, DBP, and MAP (on arrival, after 1 h, and after 24 h), the study's findings showed a statistically significant difference (*p-*value < 0.001) between the studied groups (Cases and Control). Moreover, there was a significant difference (*p-*value < 0.001) in hemoglobin, hematocrit, base deficit (both at arrival and after the first hour of resuscitation), and PH between the study groups (Cases and Control).

In our investigation, there was no statistically significant difference between the two groups (Cases and Control) with respect to Glasgow Coma Scale ( GCS) at arrival and PH following an hour of resuscitation (*p-*value 0.164, 0.161).

Luo et al.'s [27] study, which involved 1051 polytrauma cases, was carried out in four Level I trauma facilities. The majority of patients were male (75.4%), which is consistent with our findings. Additionally, there was no significant difference in gender or GCS scores between patients with shock and those who were not (p > 0.05), which is also consistent with our findings [27].

Khajehpour and Behzadnia, 2022 discovered that there was no significant difference in systolic blood pressure (RR) between the two trauma groups—the hemorrhagic shock group (n = 36) and the non-hemorrhagic shock group (n = 39). This finding contradicts our findings. Laboratory parameters (lactate level) and other clinical parameters (shock index, injury severity score) showed significant differences between the two groups (P < 0.05) [28] which is consistent with what we had found.

Furthermore In patients who arrived in the emergency room with hypovolemia as a result of trauma from an accident, Shah et al. [29] assessed the safety of polygeline (colloid) up to six hours after administration. After receiving polygeline for one hour and six hours, all vital signs (blood pressure, pulse, and respiration rate) considerably improved (p < 0.001). Additionally, at 1 and 6 h, mean blood lactate levels demonstrated a substantial decrease from the baseline (p < 0.05), which is consistent with our findings [29].

Ozakin et al.'s [30] study, which comprised 359 patients who were admitted to the ED as a result of blunt multi-trauma, concurs with our findings as well. When two groups (one that received blood transfusions and the other that did not) were compared, there was a notable difference in systolic blood pressure (SBP), Diastolic blood pressure (DBP),Heart rate.

( HR), Respiratory Rate RR, Oxygen Saturation SpO2, GCS, Hb, Htc, lactate, Base Deficit BD, pH, Shock index (*p* < 0.05 separately) [30].

### IVC ultrasound measures as a diagnostic technique for hypovolemic shock identification

In our investigation, we found a significant difference (*P-*value < 0.01*) in IVCci at baseline (time0) between hypovolemic shocked patients. This suggests that, in practice, we can utilize IVCci as a diagnostic tool to identify hypovolemic shock prior to initiating resuscitation. Furthermore, we observed a significant (*P-*value < 0.01*) difference in IVCmax0 between non-shocked individuals and hypovolemic shock patients. In the former, IVCmax was flat with mean ± SD(10.1 ± 2.6), while in the latter, it was with mean ± SD (18.3 ± 2.4).Our results concur with those of Dipti A. et al. [6], who conducted a meta-analysis of data from five research regarding the sonographic The maximal IVC diameter is lower (6.3 mm, 95% confidence interval 6–6.5 mm) in hypovolemic patients than in euvolemic patients, according to data from the IVC measurement used to evaluate the fluid status in the emergency department (ED) [6].

The validity of measuring fluid response using ultrasonography in the inferior vena cava.

We observed that IVCci0 at cut off point > 38.5 has strong 95%CI(0.938 – 1.0), sensitivity of 80.0%, and specificity of 85.71% with AUC of 0.971. These results suggest that IVCci of 38.6% or above can be used to identify fluid responsiveness and to distinguish between patients who are depleted and those who are not. We also discovered that IVCci at cutoff point > 28.6 had sensitivity of 80.0% and specificity of 71.43% after 1 h (after fluid resuscitation) with an AUC of 0.886 and a good 95% confidence interval (0.803 – 0.968). which mean that IVCci of 28.5% or less can indicate fluid unresponsiveness.

This suggests that fluid unresponsiveness may be indicated by an IVCci of 28.5% or less.

In 2022, Ismail et al. also studied all forms of shock pertaining to both inferior vena cava and central venous pressure. Of the 44 patients with hypovolemic shock, 23 (or 52.3%) responded, while 21 (36.2%) did not. IVC max diameter can identify hypovolemia (pvalue < 0.05, 0.003) because it shows a significant difference between responders and non-responders at times 0 and 30. According to their findings, IVCci0 (at baseline) at cut off point 40 has 100% sensitivity and 90% specificity, with an AUC of 0.976 and a good 95% confidence interval (0.92–1.03). This suggests that IVCci of 40% or above can be a sign of fluid responsiveness. IVCci of 40% or above can suggest fluid responsiveness, according to IVCci0 (at baseline) at cut off point 40, which has sensitivity 100% and specificity 90.5% with an AUC of 0.976 and good 95%CI (0.92–1.03). These findings support our work [31] by showing that IVC respiratory fluctuations can predict fluid responsiveness in patients with hypovolemic shock and identify which patients will benefit the most.

The similar conclusion was reached by Kent et al. in 2013, who stated that intravascular volume status and clinical response to volume resuscitation could be reliably predicted by IVCD measurements and the IVC-CI calculation. When IVC-CI > 50%, patients with hypovolemia are more likely to receive a diagnosis. If it is less than 20%, the patient can have either euvolemia or hypervolemia [32].

Our findings also align with those of the 2018 study conducted by Elbaih et al. In assessing intravascular volume among hypovolemic patients, they examined the validity of the shock index, modified shock index, central venous pressure, and inferior vena cava collapsibility index. They found that IVC-CI exhibits 100% specificity and sensitivity in diagnosing hypovolemic status when ≥ 50% [19].

Contrary to our findings, Airapetian et al. [33] discovered that in ICU patients who are breathing on their own and are suspected of having hypovolemia, vena cava size and respiratory variability do not predict fluid response. Responders, or 29 people, or 49% of the total, were those whose cardiac output increased by 10% or more following fluid infusion. Between responders and nonresponders, there were no statistically significant differences in cIVC and IVCmax at baseline. The area under the curve for cIVC at baseline was 0.62 ± 0.07, 95% confidence interval (0.49–0.74), while the area under the curve for IVCmax was 0.62 ± 0.07, 95% confidence interval (0.49–0.75), indicating that the fluid-responsiveness prediction using cIVC and IVCmax was not very accurate [33].

In Yiwen Chong et al.'s study from 2023, 31 full-term delivery women who gave birth vaginally experienced an estimated postpartum hemorrhage of at least 500 mL of blood loss. The end-expiratory (IVCe) and end-inspiratory (IVCi) diameters of the inferior vena cava were measured. IVCe IVC-CI increased significantly (31.1 ± 13.7%) when postpartum bleeding reached 500 mL in T4 compared to T3 (the third stage of labor) (27.7 ± 14.0%) (*P* = 0.005). When the IVC diameters were assessed, there was no discernible change in either HR or MAP at that same moment. (P greater than 0.05).According to these findings, IVC alterations are a more reliable indicator of low blood volume in patients suffering from postpartum hemorrhage than blood pressure and HR [34].

Similar findings were made by Zengin et al. in 2013 when they discovered that hypovolemic patients' arrival IVCe, IVCi, and dRV average diameters were considerably smaller than those of healthy volunteers (*P* = 0.001). The mean diameters of the RV, IVCe, and IVCi increased significantly in hypovolemic patients following fluid resuscitation (*P* = 0.001) [35]

However, a meta analysis conducted in 2017 by Long et al. found that respiratory variation in IVC diameter is not a reliable indicator of fluid responsiveness, especially in patients who are ventilating spontaneously. Considering the clinical context is important when utilizing IVC ultrasonography to guide therapy options. There were seventeen studies included in the analysis. The same was done in a meta-analysis for the caval index by Orso D. et al. in 2020 using 20 studies: the sensitivity, specificity, logarithmic diagnostic odds ratio, and pooled area under the curve were 0.71, 0.75, and 0.71, respectively. Furthermore, he claims that ultrasonography assessment of the IVC's width and its respiratory changes does not appear to be a trustworthy way to forecast fluid responsiveness [37].

Our investigation revealed that, after a 24-h period, the Cases (shocked) group had a significantly higher total fluid demand (*p-*value < 0.001) than the Control (non-shocked) group (1595 ± 494.8). In terms of fluid requirement at the first hour, amount of blood transfusion, and highly statistically significant (*p-*value < 0.001) increased total fluid requirement (24 h) in non-repleted cases (3571.4 ± 705.4) compared with repleted cases (2143.7 ± 397.9), there was also no statistically significant difference between repleted and non-repleted cases.

In 2020, Doucet et al. found 196 hospital admissions with IVCD of 12 mm or IVCCI of 50% or below, of which 144 were included. There were 58 non-repleted and 86 repleted. Higher IVCCI (41.7% ± 30.0% vs. 13.2% ± 12.7%, p < 0.001) and lower IVCD (6.0 ± 3.7 mm vs. 14.2 ± 4.3 mm, p < 0.001) were seen in nonrepleted individuals.yet there was no discernible change in IJVCCI or IJVD (internal jugular venous diameter). Compared to Nonrepleted, Repleted had a higher 24FR (2503 ± 1751 mL vs. 1,243 ± 1,130 mL, *p* = 0.003). IVCDMIN and IVCCI both predicted 24FR (area under the curve [AUC], 0.74; 95% confidence interval [CI], 0.64–0.84; p < 0.001), according to receiver operating characteristic analysis [12].

Patients with a large IVC volume (fourth quartile) were less likely to receive massive transfusion than those with a small IVC volume (first quartile, ≥ 28.29 mL: 0% vs < 15.08 mL: 20.3%, OR: 0.13, 95% CI, 0.03–0.66). This information was discovered in a study conducted in 2021 by Chien et al. and involving 236 trauma patients. The AUC of the IVC volume was 0.90 (95% CI, 0.81–0.99)(38) for patients who were not receiving mechanical ventilation during the CT scan, which is similarly consistent with our findings.

Innocenti et al. [39] assessed individuals with acute circulatory failure who were not ventilated and had previously had the first resuscitation. Patients who were not fluid responsive (FR) consumed considerably less fluid in the first 12 h following the examination than those who were (FR) (1119 ± 410 vs 2010 ± 1254 ml, *p* < 0.001). Both FR and non-FR patients got a similar initial fluid bolus; however, the non-FR patients had a lower dosage of maintenance infusion than the FR patients [39] which is consistent with our findings.

### Limitations of the study

Our study was conducted in a single hospital, which may limit the generalizability of our results whether Suez Canal University Hospitals serves five governorates.

The study population was relatively small; therefore, we cannot draw concrete conclusions.

## Conclusion

The assessment of hemodynamic status in polytrauma patients is an important principle of the primary survey of trauma patients Our study showed that Repeated bedside ultrasonography of IVCD, IVCci before and after the first hour of resuscitation could be a good, reliable invasive tool that can be used in estimating First 24 h fluid requirement in Major blunt trauma patients and assessment of fluid status.We can say that IVCci of 38.5% or more is a good prediction of fluid responsiveness. These results are valuable for hypovolemic patients.

## Recommendations

From the study results we recommend:

- Repeated IVC ultrasound for hypovolemic unstable patients and continuous monitoring to the fluid responsiveness in loading and maintenance fluid and prediction of 24 h fluid requirement.
- Ultrasound devices should be available in each emergency department.
- All emergency physicians should have point-of-care ultrasound as a part of their training course.

## Data Availability

All data analyzed during this study are included in this published research.

## Abbreviations

IVC: Inferior vena cava
Ivc-ci: Inferior vena cava collapsibility index
IVCD: Inferior Vena Cava Diameter, (Diameter Inferior Vena Cava at Minimum of inspiration) and
DIVC Max: Diameter Inferior Vena Cava at Maximum of expiration
DIVC Min: Diameter Inferior Vena Cava at Minimum of inspiration
AUC: Area under the ROC Curve
CVP: Central venous pressure
FAST: Focused assessment with sonography for trauma
ISS: Injury Severity Score
RA: Right Atrium
NPV: Negative predictive value
PPV: Positive predictive value
GCS: Glasgow Coma Scale
